# P-1836. SARS-CoV-2 induces blood-brain barrier and choroid plexus barrier impairments and vascular inflammation in mice

**DOI:** 10.1093/ofid/ofae631.1999

**Published:** 2025-01-29

**Authors:** Haowen Qiao

**Affiliations:** University of Southern California, Los Angeles, California

## Abstract

**Background:**

The coronavirus disease of 2019 (COVID-19) pandemic has led to more than 700 million confirmed cases and nearly 7 million deaths. Although Severe Acute Respiratory Syndrome Coronavirus-2 (SARS-CoV-2) virus mainly infects the respiratory system, neurological complications are widely reported in both acute infection and long-COVID cases. Despite the success of vaccines and antiviral treatments, neuroinvasiveness of SARS-CoV-2 remains an important question, which is also centered on the mystery whether the virus is capable of breaching the barriers into the central nervous system.

**Figure 1:**
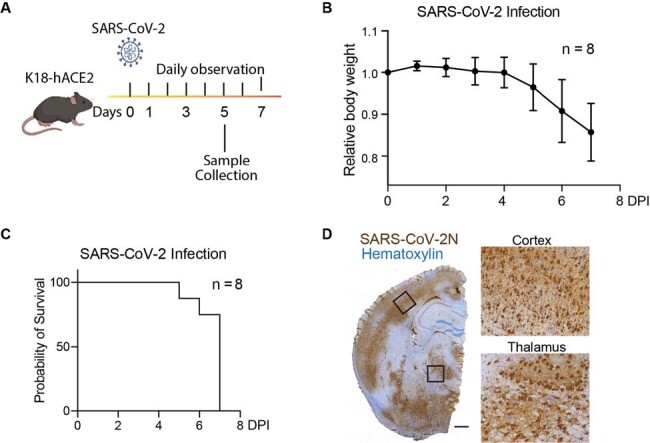
SARS-CoV-2 infection in the K18-hACE2 model. (A) Experimental diagram: Eight weeks-old K18-hACE2 mice were intranasally infected with SARS-CoV-2, monitored daily for symptoms, or euthanized at 5 days post-infection (DPI) to collect tissue. (B) Daily weight measurements in K18-hACE2 mice intranasally infected with SARS-CoV-2 isolate USA-WA1/2020. (C)The survival curve showing the probability of survival over 7 days after SARS-CoV-2 infection. (D) Representative mouse brain hemisphere image showing the presence of SARS-CoV-2 nucleocapsid protein by immunohistochemical staining. Boxed regions are shown on the right. Bar: 500 µm.

**Methods:**

Here using the K18-hACE2 model, we investigated the impact of acute SARS-CoV-2 infection on major parts of neurovascular systems, and found increased incidence of microhemorrhage and significant disruption of both BBB and BCSFB in K18-hACE2 mice after SARS-CoV-2 infection. Cerebral microvascular injury was accompanied by substantial pericyte damage, tight junction loss, astrogliosis, and neuroinflammation in the brain parenchyma. In addition, endothelial activation and vascular inflammation occurred at both BBB and BCSFB, as shown by upregulation of VCAM-1 and COX2 markers.

**Figure 2:**
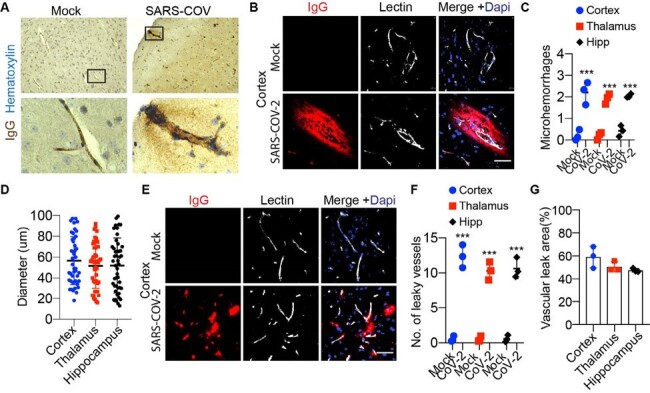
Vascular damage and BBB breakdown in SARS-CoV-2 infected K18-hACE2 model. (A) Representative images showing IgG immunohistochemical staining in K18-hACE2 mouse brain tissues with or without SARS-CoV-2 infection. Boxed regions are shown at the bottom. (B) Representative images showing immunofluorescent staining with IgG, showing the microhemorrhage site in the cortex of K18-hACE2 mice with SARS-CoV-2 infection. Bar: 50 µm. (C) Quantification of the number of microhemorrhages per field of view in the cortex, thalamus, and hippocampus (Hipp). n= 3; ***p< 0.001; two-tailed Student’s t-test. (D) Quantification of the diameters of the microhemorrhages in the cortex, thalamus, and hippocampus. (E) Representative images showing immunofluorescent staining for IgG at the capillary level in the cortex. K18-hACE2 mice with SARS-CoV-2 infection exhibited a significant accumulation of IgG surrounding the microvessels. Bar: 50 µm. (F) Quantification of the number of leaky small blood vessels per field of view in cortex, thalamus, and hippocampus. n= 3; ***p< 0.001; two-tailed Student’s t-test. (G) Quantification of the percentage of leakage vascular area to the total area of blood vessels in cortex, thalamus, and hippocampus. n= 3.

**Results:**

By studying the K18-hACE2 infection model, we observed clear evidence of microvascular damage and breakdown of the blood-brain barrier (BBB). Mechanistically, SARS-CoV-2 infection caused pericyte damage, tight junction loss, endothelial activation and vascular inflammation, which together drive microvascular injury and BBB impairment.

BBB tight junction loss in SARS-CoV-2 infected K18-hACE2 model.

**Figure:**
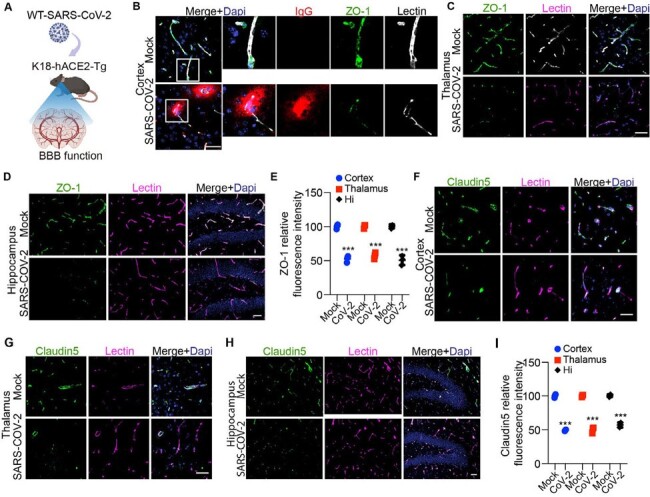

(A) The K18-hACE2 mice were used to test the BBB function after SARS-CoV-2 infection. (B) Representative images showing immunofluorescent staining for IgG, ZO-1, and Lectin in the cortex of K18-hACE2 mice. Bar: 50 µm. (C-D) Representative images showing immunofluorescent staining for ZO-1 and Lectin in the thalamus(C) and hippocampus(D) areas of K18-hACE2 mice. Bar: 50 µm. (E) Length of ZO-1-positive profiles in the cortex, thalamus, and Hipp. n= 3; ***p< 0.001; two-tailed Student’s t-test. (F-H) Representative images showing immunofluorescent staining for Claudin5 and Lectin in the cortex(F), thalamus (G), and hippocampus (H) areas of K18-hACE2 mice. Bar: 50 µm. (I) Claudin5 length in the cortex, thalamus and, Hipp. n= 3; ***p< 0.001; two-tailed Student’s t-test.

**Conclusion:**

The impact of such changes, together with astrogliosis and neuroinflammation, may drive or at least contribute to the neurological complications seen in COVID-19 patients. As SARS-CoV-2 will likely remain a major health issue for years to come, our findings provide a needed understanding of its impact on the major CNS barriers and brain homeostasis at both molecular and cellular levels.

Vascular inflammation in SARS-CoV-2 infected K18-hACE2 model

**Figure:**
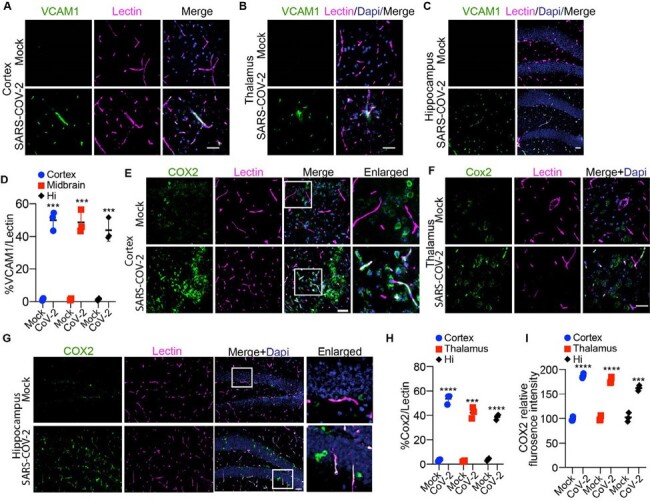

(A-C) Representative images showing immunofluorescent staining for VCAM1 and Lectin in the cortex, thalamus, and hippocampus of K18-hACE2 mice. Bar: 50 µm. (D) Quantification of VCAM1 and lectin signal overlap in the cortex, thalamus, and hippocampus. n= 3; ***p< 0.001; two-tailed Student’s t-test. (E-G) Representative images showing immunostaining of COX2 and Lectin in the cortex, thalamus, and hippocampus of K18-hACE2 mice. Bar: 50 µm. (H) Quantification of COX2 and Lectin signal overlap in the cortex, thalamus, and hippocampus areas of K18-hACE2 mice. n= 3; ***p< 0.001; ****p< 0.0001; two-tailed Student’s t-test. (I) Quantification of COX2 relative fluorescence intensity in the cortex, thalamus, and hippocampus of K18-hACE2 mice. n= 3; ***p< 0.001; ****p< 0.0001; two-tailed Student’s t-test.

**Disclosures:**

**All Authors**: No reported disclosures

